# Effect of home cook interventions for salt reduction in China: cluster randomised controlled trial

**DOI:** 10.1136/bmj-2022-074258

**Published:** 2023-08-24

**Authors:** Xiaochang Zhang, Puhong Zhang, Danyang Shen, Yuan Li, Feng J He, Jixiang Ma, Wei Yan, Yifu Gao, Donghui Jin, Ying Deng, Fangming Guo, Shichun Yan, Jing Song, Graham A MacGregor, Jing Wu

**Affiliations:** 1 Chinese Center for Disease Control and Prevention, Beijing, China; 2 The George Institute for Global Health, Beijing, China; 3 The George Institute for Global Health, Faculty of Medicine, University of New South Wales, Sydney, Australia; 4 Beijing Center for Diseases Control and Prevention, Beijing, China; 5 Wolfson Institute of Population Health, Barts and The London School of Medicine and Dentistry, Queen Mary University of London, London, UK; 6 Shandong Center for Disease Control and Prevention, Jinan, China; 7 Jiangxi Center for Disease Control and Prevention, Nanchang, China; 8 Hebei Center for Disease Control and Prevention, Shi Jiazhuang, China; 9 Hunan Center for Disease Control and Prevention, Changsha, China; 10 Sichuan Center for Disease Control and Prevention, Chengdu, China; 11 Qinghai Center for Disease Control and Prevention, Xining, China; 12 Heilongjiang Center for Disease Control and Prevention, Haerbin, China; 13 National Center for Chronic and Noncommunicable Disease Control and Prevention, Chinese Center for Disease Control and Prevention, Beijing, China

## Abstract

**Objective:**

To determine the effects of salt reduction interventions designed for home cooks and family members.

**Design:**

Cluster randomised controlled trial.

**Setting:**

Six provinces in northern, central, and southern China from 15 October 2018 to 30 December 2019.

**Participants:**

60 communities from six provinces (10 communities from each province) were randomised; each community comprised 26 people (two people from each of 13 families).

**Interventions:**

Participants in the intervention group received 12 month interventions, including supportive environment building for salt reduction, six education sessions on salt reduction, and salt intake monitoring by seven day weighed record of salt and salty condiments. The control group did not receive any of the interventions.

**Main outcome measure:**

Difference between the two groups in change in salt intake measured by 24 hour urinary sodium during the 12 month follow-up.

**Results:**

1576 participants (775 (49.2%) men; mean age 55.8 (standard deviation 10.8) years) from 788 families (one home cook and one other adult in each family) completed the baseline assessment. After baseline assessment, 30 communities with 786 participants were allocated to the intervention group and 30 communities with 790 participants to the control group. During the trial, 157 (10%) participants were lost to follow-up, and the remaining 706 participants in the intervention group and 713 participants in the control group completed the follow-up assessment. During the 12 month follow-up, the urinary sodium excretion decreased from 4368.7 (standard deviation 1880.3) mg per 24 hours to 3977.0 (1688.8) mg per 24 hours in the intervention group and from 4418.7 (1973.7) mg per 24 hours to 4330.9 (1859.8) mg per 24 hours in the control group. Compared with the control group, adjusted mixed linear model analysis showed that the 24 hour urinary sodium excretion in the intervention group was reduced by 336.8 (95% confidence interval 127.9 to 545.7) mg per 24 hours (P=0.002); the systolic and diastolic blood pressures were reduced by 2.0 (0.4 to 3.5) (P=0.01) and 1.1 (0.1 to 2.0) mm Hg (P=0.03), respectively; and the knowledge, attitude, and behaviours in the intervention group improved significantly.

**Conclusions:**

The community based salt reduction package targeting home cooks and family members was effective in lowering salt intake and blood pressure. This intervention has the potential to be widely applied in China and other countries where home cooking remains a major source of salt intake.

**Trial registration:**

Chinese Clinical Trial Registry ChiCTR1800016804.

## Introduction

Excess dietary salt consumption is a major risk factor for hypertension, stroke, heart disease, kidney disease, and gastric cancer.^1^ ^2^ ^3^ ^4^ High salt intake is the third leading risk factor and the most important dietary risk factor contributing to death and disability adjusted life years in China.^5^

Salt reduction has been adopted as one of the most cost effective public health policies worldwide. The World Health Organization and the United Nations Food and Agriculture Organization issued a joint report in 2003, calling for a reduction in population salt intake to less than 5 g per day (<2000 mg sodium).^6^ However, salt intake in the Chinese population is more than double the recommended limit.^7^ In China, most dietary salt (76%) comes from salt added during home cooking,^8^ differing from western countries where dietary salt mainly comes from manufactured food.^9^ The Chinese government has been making great efforts in salt reduction campaigns, such as mass media campaign and the distribution of salt spoons and jars to families as a reminder of salt reduction for home cooks.^10^ ^11^ ^12^ However, the effectiveness of those interventions has not been evaluated by a well designed randomised controlled trial using 24 hour urinary sodium excretion, which is the gold standard method of assessing dietary salt intake.^13^

As salt used in home cooking is the major contributor to salt intake in Chinese families, and home cooks are the people responsible for purchasing food and preparing meals for family members, comprehensive interventions should be provided for home cooks, and the effectiveness should be critically evaluated. In addition, few randomised controlled trials have been conducted to examine the effect of salt reduction interventions focusing on health education and behaviour change in the community setting in China.^14^ Hence, we developed a package of salt reduction interventions for home cooks and family members and aimed to evaluate its effectiveness in communities.

## Methods

### Study design

A parallel cluster randomised controlled trial was conducted between October 2018 and December 2019 in communities from six cities in six provinces—Qinghai, Hebei, Heilongjiang, Sichuan, Jiangxi, and Hunan. A detailed design has been published elsewhere.^15^ In summary, we selected 10 communities (clusters) with similar population sizes and similar distributions in age, economic level, and health service resources from each province and randomised them to intervention and control groups.

### Participants

Twenty six adults from 13 families, two from each including the usual cook, were recruited from each community for outcome evaluation. Eligible people were aged between 18 and 75 years and were home cooks and family members who ate homemade meals at least four times every week. If more than one family member in a family agreed to participate in the outcome assessments, the spouse of the home cook or a family member of the opposite sex to the home cook would be selected first. Participants resided in the community for more than six months and had no relocation plans for the next 24 months. We excluded anyone who was pregnant or lactating or could not collect or refused to collect 24 hour urine.

### Interventions

The intervention included health education lectures, monitoring of salt intake, and establishment of a supportive environment. The intervention lasted for one year.

The lectures comprised six sessions of education on salt reduction, disseminating knowledge on the harmful effects of high sodium intake on health, the source of salt in the daily diet, use of low sodium salt, skills for reducing salt intake during cooking (for example, using natural spices instead of salty condiments to improve the taste) and tips for purchasing pre-packaged food (for example, choosing low sodium products by reading nutrition labels). Each lesson lasted 40 minutes, and lessons were conducted once every two months by trained personnel from the local county Center for Disease Control and Prevention (CDC). From session two to session six, a quiz was used before the lecture to help participants to review the key points taught in the previous sessions. Home cooks were invited to participate in all the lectures (and family members were also encouraged to do so).

Salt intake was monitored by using a seven day estimation method.^14^ Every participant received a form that required a seven day diary on salt intake to be completed. On the first day and the seventh day, participants were instructed by the personnel from the local CDC or primary healthcare centre to weigh and record salt and other condiments used in home cooking. They were also asked to record the frequency of eating out during the seven days and the consumption of processed food for each meal. All record forms were then gathered by local workers and a WeChat app was applied to calculate the average salt consumption through the embedded algorithm. Salt reduction advice was also generated for each family member according to their salt intake and the major sources of salt in their diet. Participants were asked to repeat this procedure every two months during the one year trial, guided by local workers.

A supportive environment containing health education information on salt reduction was established in the community through various media strategies, including posters, short videos, loudspeaker broadcasts, leaflets, and manuals. In addition, we provided salt restriction spoons to the families as practical tools to reduce salt intake. The intervention aimed to create an atmosphere of salt reduction all over the community, so as to help to promote individual behaviour change. A detailed description of the intervention activities is provided in supplementary table A.

### Outcomes

The primary outcome was the difference between the two groups in the change from baseline to follow-up in salt intake measured by 24 hour urinary sodium. The secondary outcomes were the differences in the change in blood pressure and in knowledge, attitude, and behaviours between the two groups.

Data were collected by well trained field investigators through a specially designed mobile device based electronic data capture system (mEDC, Electronic Data Collection). The urine sample was excluded if the collection time was less than 20 hours or more than 28 hours. If the duration of urine collection was not 24 hours but within 20-28 hours, we calculated adjusted 24 hour urine volume as total volume divided by collection time and multiplied by 24. The calculations of 24 hour urinary sodium and potassium were as follows: sodium (mg/day)=23 (mg/mmol)×concentration (mmol/L)×adjusted 24 hour urine volume (L/day); potassium (mg/day)=39.1 (mg/mmol)×concentration (mmol/L)×adjusted 24 hour urine volume (L/day).

The biochemists who made the measurements of urinary electrolytes were unaware of the group to which the participant was allocated. Urine samples were defined as incomplete and excluded from the analysis if the 24 hour urine volume was <500 mL or creatinine was <4.0 mmol for women or <6.0 mmol for men.^16^ Trained personnel measured blood pressure by using Omron HEM-7125 electronic sphygmomanometers in a separate room. Before the measurement, the participants should sit quietly for at least five minutes. Blood pressure was calculated using the mean of the last two of the three measurements. We defined hypertension as mean systolic blood pressure ≥140 mm Hg, mean diastolic blood pressure ≥90 mm Hg, or self-reported use of antihypertensive drugs in the previous two weeks.^17^

### Sample size

We assumed a standard deviation for 24 hour urinary sodium excretion of 85 mmol/24 hours (1960 mg/day) and an intra-class correlation coefficient of 0.05.^18^ On the basis of these assumptions, we estimated that a sample size of 1426 people (713 home cooks and 713 family members) could provide a power of 80% (with a two sided α of 0.05) to detect a difference in mean 24 hour urinary sodium ≥20 mmol/day (460 mg/day or 1.15 g/day salt) between the two groups. We took a 20% dropout rate of participants into account. In addition, considering that the response rate for 24 hour urine collection is low in many cases,^19^ we added at least one family per community, resulting in the recruitment of at least 1560 people (780 home cooks and 780 family members) for outcome assessment.

### Randomisation and masking

The 60 communities were assigned to the intervention group or the control group in a one to one ratio. Randomisation was computer generated centrally by the Chinese CDC, stratified by provinces. The randomisation procedure was carried out after the baseline survey, and the participants and local investigators who undertook recruitment and baseline data collection were unaware of the group allocation.

### Statistical analysis

We analysed the data according to the intention-to-treat principle. Participants who completed the baseline survey were analysed according to their randomly assigned group. We compared the difference in 24 hour urinary sodium excretion, as well as the secondary outcomes, between the two groups by using linear mixed models with participants nested within family units and families nested within community/village units. We included groups (intervention and control), time (baseline and end of the trial), (time×group) interactions, and potential confounding variables, including age, sex, body mass index, province, and education level, in the model, with the (time×group) interaction term indicating different changes by the group from baseline to the end of the trial. We did post hoc subgroup analyses considering age, sex, education level, family income, and hypertension status. To test the robustness of the primary findings, we did two sensitivity analyses: analysing the results without adjustment for the potential confounding variables and adding possibly incomplete 24 hour urine collections to the samples for analysis.

We used SAS version 9.4 for the analyses and calculated all P values as two sided. We determined statistical significance by a false discovery rate of less than 0.05.

### Patient and public involvement

Provincial CDCs, county level CDCs, and primary healthcare centres were involved in the design and conduct of this study. At the protocol stage, we gained their opinions on the content of the intervention and the electronic data collection system. Personnel at the county level CDCs helped to translate the educational materials, which were produced by the research team, into language appropriate to the local people.

## Results

**Fig 1:**
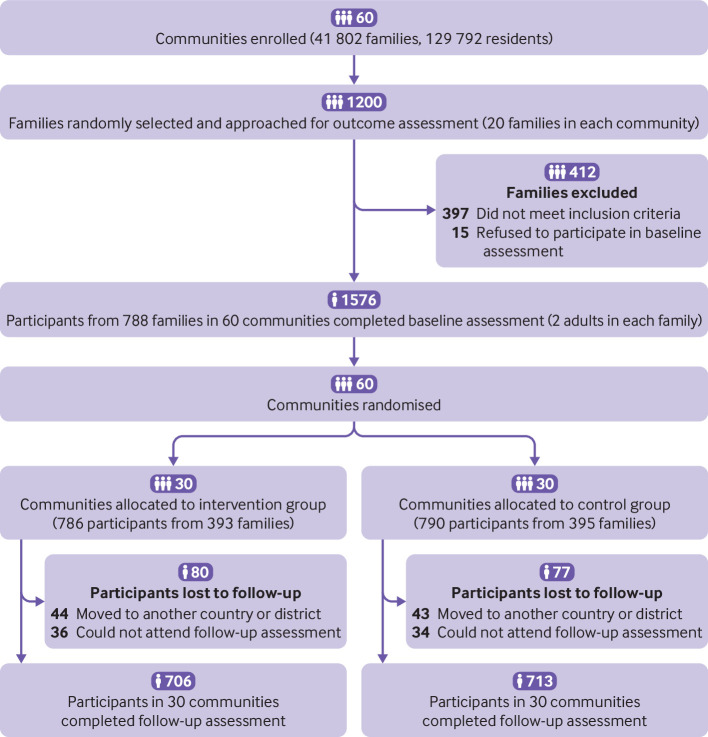
Flowchart of participants

We recruited 60 communities (10 in each province) into the study in October 2018. A total of 1576 participants from 788 families completed the baseline survey. The mean age was 55.8 (standard deviation 10.8) years, and 775 (49.2%) were male. During the trial, 157 (10%) participants were lost to follow-up as they moved to another county or were unable to attend follow-up assessments (fig 1). The characteristics of participants lost to follow-up are described in supplementary table B. Table 1 shows that the baseline characteristics of participants in the two groups were similar.

**Table 1:** Baseline characteristics. Values are numbers (percentages) unless stated otherwise

Characteristics	Control (n=790)	Intervention (n=786)
**Cluster level**		
No of communities by location:		
Hebei	5/60	5/60
Heilongjiang	5/60	5/60
Jiangxi	5/60	5/60
Hunan	5/60	5/60
Sichuan	5/60	5/60
Qinghai	5/60	5/60
No of families	395	393
Mean (SD) outdoor temperature (°C)	7.9 (13.5)	12.1 (6.9)
Total No of residents in communities	63 026	66 766
Male gender	32 498 (51.6)	33 941 (50.8)
Age, years:		
18-44	17 901 (36.2)	19 362 (38.1)
45-59	19 874 (40.2)	19 225 (37.8)
60-75	11 621 (23.5)	12 286 (24.2)
Mean (SD) No of primary care providers in each community	3.1 (2.9)	2.5 (2.3)
**Individual level**		
Male gender	391 (49.5)	384 (48.9)
Mean (SD) age, years	55.0 (10.9)	56.6 (10.5)
Education level:		
Never enrolled in education	177 (22.4)	169 (21.5)
Primary school	232 (29.4)	246 (31.3)
Junior high school	259 (32.8)	234 (29.8)
High school and above	122 (15.4)	137 (17.4)
Total annual household income, yuan:	(n=698)	(n=708)
≤25 000	364 (52.1)	380 (53.7)
25 000	334 (47.9)	328 (46.3)
Current alcohol drinking	294 (37.2)	296 (37.7)
Physical activity	219 (27.7)	249 (31.7)
Mean (SD) body mass index	25.1 (7.6)	25.1 (3.8)

SD=standard deviation.

The establishment of a supportive environment intervention covered all the people living in the intervention communities. Data collected during the intervention showed that 953 posters were put up, and 10 510 leaflets and 5265 manuals were distributed to people living in the intervention communities. Short videos were played in the health service stations of 25 communities in five provinces (not in Qinghai). Loudspeaker broadcasts were played in 25 communities in five provinces (not in Sichuan). Six lectures and six salt intake monitoring activities were organised in all communities in the intervention group as planned. The number of people who took part in the lectures was 4975 (on average, 28 for each lecture in each community).

The mean baseline urinary sodium excretion was 4418.7 (standard deviation 1973.7) mg per 24 hours in the control group and 4368.7 (1880.3) mg per 24 hours in the intervention group. During the 12 month trial, we observed decreases in sodium excretion in the two groups after adjustment for confounders. After adjustment, the mean difference between the intervention group and the control group was −336.8 (95% confidence interval −545.7 to −127.9) mg per 24 hours, which is equivalent to a 0.9 g per 24 hours decrease in salt intake (table 2). Blood pressure also decreased in both groups, and to a greater extent in the intervention group. The mean effect was −1.98 (−3.54 to −0.41) mm Hg for systolic pressure and −1.05 (−2.01 to −0.10) mm Hg for diastolic pressure (table 2). The subgroup analyses showed no significant difference in the mean effect on urinary sodium or blood pressure between genders, age groups, education level, family income, hypertension, and family role (table 3, supplementary tables C and D).

**Table 2:** Results for 24 h urinary sodium excretion, potassium excretion, sodium-to-potassium ratio, and blood pressure

Outcomes	Control							Intervention							Intervention *v* control	
	Baseline			12 months		Change from baseline* † (95% CI)		Baseline			12 months		Change from baseline* † (95% CI)		Difference in change* ‡ (95% CI)	P value	
	No	Mean (SD)		No	Mean (SD)			No	Mean (SD)		No	Mean (SD)					
Salt, g/24 h	769	11.2 (5.0)		678	11.0 (4.7)	−0.1 (−0.5 to 0.3)		761	11.1 (4.8)		667	10.1 (4.3)	−1.0 (−1.3 to −0.6)		−0.9 (−1.4 to −0.3)	0.002	
Urinary sodium, mg/24 h	769	4418.7 (1973.7)		678	4330.9 (1859.8)	−40.8 (−188.7 to 107.1)		761	4368.7 (1880.3)		667	3977.0 (1688.8)	−377.6 (−526.3 to −228.9)		−336.8 (−545.7 to −127.9)	0.002	
Urinary potassium, mg/24 h	769	1578.5 (617.0)		678	1513.0 (598.6)	−74.1 (−125.9 to −22.3)		761	1593.8 (616.3)		667	1542.4 (599.0)	−59.6 (−11.6 to −7.5)		14.5 (−58.6 to 87.6)	0.70	
Sodium-to-potassium ratio, mmol/mmol	769	5.07 (2.20)		678	5.26 (2.29)	0.27 (0.09 to 0.44)		761	4.98 (2.16)		667	4.73 (2.12)	−0.21 (−0.39 to −0.03)		−0.47 (−0.72 to −0.22)	<0.001	
Systolic blood pressure, mm Hg	790	128.9 (19.1)		713	130.5 (19.2)	−0.03 (−1.20 to 1.15)		785	129.9 (19.14)		706	130.1 (18.16)	−2.00 (−3.40 to −0.61)		−1.98 (−3.54 to −0.41)	0.01	
Diastolic blood pressure, mm Hg	790	80.1 (10.8)		713	80.1 (11.0)	−0.34 (−1.06 to 0.37)		785	80.1 (11.27)		706	79.3 (11.28)	−1.40 (−2.24 to −0.55)		−1.05 (−2.01 to −0.10)	0.03	

CI=confidence interval; SD=standard deviation.

* Results were obtained from mixed linear model with random intercept of participants nested within family and random intercept of family nested within communities; all values for urinary outcomes were adjusted for age, sex, body mass index at baseline and follow-up, province, and education level. All values for blood pressure were further adjusted for outdoor temperature at baseline and follow-up, physical activity, and alcohol drinking status.

† Comparison of means within each group. Positive values indicate increase from baseline to 12 month follow-up; negative values indicate decrease from baseline to 12 month follow-up.

‡ Comparison between intervention group and control group in changes from baseline to 12 month follow-up. Positive values indicate that intervention group had greater increase or smaller decrease from baseline to 12 month follow-up compared with control group; negative values indicate that intervention group had greater decrease or smaller increase from baseline to 12 month follow-up compared with control group.

**Table 3:** Results for 24 hour urinary sodium excretion by subgroup (mg/24 h)

Outcomes	Control							Intervention							Intervention *v* control		P for interaction
	Baseline			12 months		Change from baseline* † (95% CI)		Baseline			12 months		Change from baseline* † (95% CI)		Difference in change* ‡ (95% CI)	P value	
	No	Mean (SD)		No	Mean (SD)			No	Mean (SD)		No	Mean (SD)					
Gender:																	0.55
Male	380	4711.6 (2026.6)		325	4599.5 (2032.2)	−77.9 (−289.4 to 133.5)		370	4630.8 (1948.4)		317	4290.0 (1832.5)	−349.0 (−563.0 to −135.1)		−271.1 (−571.4 to 29.3)	0.08	
Female	389	4132.5 (1879.5)		353	4083.6 (1650.3)	−9.1 (−214.1 to 195.9)		391	4120.8 (1780.7)		350	3693.4 (1494.1)	−407.1 (−612.5 to −201.6)		−397.9 (−687.7 to −108.2)	0.007	
Age, years§:																	0.11
Quarter 1 (18-49.1)	227	4529 (147)		156	4755 (166)	225.7 (−102 to 553.2)		186	4710 (160)		144	4096 (177)	−613.3 (−959 to-267.8)		−239.1 (−618 to 140)	0.22	
Quarter 2 (49.2-56.4)	204	4598 (123)		174	4535 (125)	−63.4 (−312 to 184.9)		194	4497 (122)		150	3993 (129)	−503.7 (−755 to-252.2)		−321.7 (−620 to-23)	0.04	
Quarter 3 (56.5-65.1)	169	4488 (123)		164	4209 (125)	−278.6 (−528 to −29.1)		199	4318 (118)		177	4015 (122)	−303.0 (−541 to −65.3)		−181.5 (−474 to111)	0.22	
Quarter 4 (65.2-75)	169	3876 (146)		184	3842 (140)	−34.1 (−323 to 254.5)		182	3878 (142)		196	3720 (137)	−157.9 (−431 to 115.7)		−60.7 (−394 to 273)	0.72	
Education level:																	0.80
Primary school and below	398	4436.3 (2021.6)		366	4347.6 (1816.4)	−18.9 (−270.1 to 232.3)		398	4409.2 (1764.3)		354	4110.7 (1692.1)	−295.7 (−501.6 to −89.7)		−120.9 (−421.1 to 179.4)	0.43	
Junior high school and above	371	4399.8 (1923.6)		312	4311.3 (1912.3)	−105.6 (−323.1 to 112.0)		363	4324.3 (2001.3)		313	3825.7 (1674.9)	−472.7 (−689.8 to −255.6)		−177.0 (−478.1 to 124.00)	0.25	
Family income, yuan:																	0.72
≤25 000	351	4571.8 (2099.1)		281	4443.4 (1894.4)	−116.7 (−353.7 to 120.4)		366	4531.8 (1909.5)		296	4103.1 (1729.5)	−475.0 (−707.5 to −242.6)		−358.4 (−689.6 to −27.1)	0.03	
>25 000	328	4279.2 (1865.0)		380	4218.3 (1821.7)	36.2 (−191.2 to 263.5)		320	4120.1 (1857.9)		351	3921.4 (1650.0)	−234.3 (−463.8 to −4.8)		−270.5 (−592.5 to 51.5)	0.10	
Hypertension:																	0.57
Yes	307	4668.4 (2165.1)		299	4465.3 (1954.3)	−137.1 (−368.0 to 93.9)		361	4577.8 (1953.2)		308	4147.3 (1686.6)	−383.8 (−604.7 to −162.9)		−248.9 (−568.2 to 70.3)	0.13	
No	462	4252.8 (1819.1)		379	4224.9 (1777.2)	17.2 (−180.0 to 214.4)		400	4180.1 (1793.6)		359	3830.8 (1679.2)	−359.2 (−566.8 to −151.6)		−376.5 (−662.2 to −90.8)	0.01	
Family role:																	0.87
Home cook	384	4208.5 (1919.1)		349	4146.6 (1682.1)	−47.1 (−253.9 to 159.7)		382	4187.3 (1768.2)		341	3758.5 (1536.4)	−400.0 (−608.3 to −191.7)		−352.9 (−646.0 to −59.8)	0.02	
Other family members	385	4628.3 (2007.3)		329	4526.4 (2015.7)	−35.7 (−246.7 to 175.3)		379	4551.6 (1972.4)		326	4205.5 (1808.9)	−356.6 (−568.3 to −144.8)		−318.2 (−616.5 to −19.8)	0.04	

CI=confidence interval; SD=standard deviation.

* Results were obtained from mixed linear model with random intercept of participants nested within family and random intercept of family nested within communities; all values for urinary outcomes were adjusted for age, sex, body mass index at baseline and follow-up, city, and education level.

† Comparison of means within each group. Positive values indicate increase from baseline to 12 month follow-up; negative values indicate decrease from baseline to 12 month follow-up.

‡ Comparison between intervention group and control group in changes from baseline to 12 month follow-up. Positive values indicate that intervention group had greater increase or smaller decrease from baseline to 12 month follow-up compared with control group; negative values indicate that intervention group had greater decrease or smaller increase from baseline to 12 month follow-up compared with control group.

§ Age was defined as natural cubic spline with internal knots at 25th, 50th, and 75th centiles.

Table 4 shows the results for knowledge, attitude, and behaviours in relation to salt reduction. After the 12 month trial, the intervention effect was significant for increasing the proportion of people with knowledge of the salt intake recommended by the Chinese nutrition guidelines (odds ratio 17.42, 95% confidence interval 11.32 to 26.82) and who had heard about a low sodium salt substitute (8.73, 5.88 to 12.97), had the ability to identify salt content on a food label (9.84, 6.67 to 14.50), were willing to choose a low sodium diet (2.86, 1.79 to 4.56), preferred a less salty taste (2.24, 1.60 to 3.13), used a low sodium salt substitute (3.66, 1.79 to 7.49), and ate processed food once a week or less (1.66, 1.23 to 2.25) .

**Table 4:** Results for knowledge, attitude, and behaviours

Outcomes	Control (n=790)				Intervention (n=786)				Intervention effect	
	No (%) at baseline	No (%) at 12 months (n=713)	Change from baseline*: OR (95% CI)		No (%) at baseline	No (%) at 12 months (n=706)	Change from baseline*: OR (95% CI)		Intervention *v* control*: OR (95% CI)	P value
**Knowledge**										
Knowledge of salt intake recommended by Chinese nutrition guidelines	119 (15.1)	152 (21.3)	1.68 (1.25 to 2.27)		137 (17.4)	535 (75.8)	29.34 (21.41 to 40.20)		17.42 (11.32 to 26.82)	<0.001
Having heard about low sodium salt substitute	141 (17.8)	219 (30.7)	2.54 (1.93 to 3.35)		169 (21.5)	552 (78.2)	22.18 (16.58 to 29.67)		8.73 (5.88 to 12.97)	<0.001
Having ability to identify salt content on food label	240 (30.4)	221 (31.0)	1.15 (0.88 to 1.49)		255 (32.4)	505 (71.5)	11.28 (8.47 to 15.04)		9.84 (6.67 to 14.50)	<0.001
**Attitude**										
Willing to choose low sodium diet	627 (79.4)	582 (81.6)	1.18 (0.89 to 1.57)		644 (81.9)	658 (93.2)	3.38 (2.33 to 4.90)		2.86 (1.79 to 4.56)	<0.001
Preferring less salty taste	244 (30.9)	235 (33.0)	1.06 (0.83 to 1.35)		211 (26.8)	314 (44.5)	2.37 (1.88 to 3.00)		2.24 (1.60 to 3.13)	<0.001
**Behaviours**										
Using low sodium salt substitute†	40 (28.4)	37 (16.9)	0.61 (0.35 to 1.06)		35 (20.7)	142 (25.7)	2.23 (1.41 to 3.54)		3.66 (1.79 to 7.49)	<0.001
Eating processed food once a week or less	426 (53.9)	386 (54.1)	1.01 (0.82 to 1.25)		423 (53.8)	463 (65.6)	1.68 (1.35 to 2.09)		1.66 (1.23 to 2.25)	0.001

CI=confidence interval; OR=odds ratio.

* Results were obtained from mixed effect logistic regression model with random intercept of participants nested within family and random intercept of family nested within communities; all values were adjusted for age, sex, body mass at baseline and follow-up, city, and education level.

† Questionnaires about using low sodium salt substitutes were filled out by people who had heard about low sodium salt substitutes.

Supplementary tables E and F show the results of the sensitivity analyses. The mean effects on urinary sodium (−318.4 (−527.0 to −109.7) mg per 24 hours) and blood pressure (systolic −1.3 (−2.8 to 0.2) mm Hg; diastolic −0.7 (−1.7 to 0.2 ) mm Hg) were smaller if not adjusted for the confounders (supplementary table E). The mean effect was similar to the main results when possibly incomplete 24 hour urine collections were included (supplementary table F).

## Discussion

We developed a set of community based salt reduction interventions targeting home cooks and family members. This cluster randomised controlled trial showed that the intervention package not only effectively reduced the urinary sodium excretion by 336.8 mg/day (0.9 g/day salt reduction) but also lowered systolic blood pressure by 1.98 mm Hg and diastolic blood pressure by 1.05 mm Hg within one year. Salt related knowledge, attitude, and behaviours also improved significantly.

Population level multicomponent salt reduction activities have the potential to reduce dietary salt intake.^20^ In particular, community based health education interventions have been shown to be an important and effective way to change the behaviours of the target population.^21^ In this study, we implemented health education interventions at both the community level and the individual level. At the community level, a supportive environment was established through various media strategies, including posters, videos, loudspeaker broadcasts, leaflets, and brochures, to spread the knowledge of and develop skills in salt reduction via visual and auditory stimulations. However, videos could not directly reach people living in remote rural areas such as Qinghai with no access or equipment to play the video, and loudspeaker broadcasts were difficult to implement in urban areas in Sichuan. The diverse health communication meant that, overall, the strategy achieved high coverage. At the individual level, lectures with interactive activities were carried out. Home cooks in the intervention group were required to participate in each lecture. Family support could play an important role in shaping the behaviours of family members to reduce salt intake.^22^ A previous study suggested that the effects of cooking classes on salt reduction for housewives could be vital for their family members and could help to reduce the salt intake of the whole family.^23^ Educating family members through various routes such as mass media, primary health providers, and home cooks, on the risks of excessive salt consumption and raising awareness of their daily salt intake and sources is also important. This will largely reduce the resistance from family members when the home cooks trying to use less salt and increase the adherence of the whole family to salt reduction.^24^ Consistently, results of this study showed that both home cooks and family members experienced a significant decrease in urinary sodium. The observed improvements in knowledge, attitude, and behaviours related to sodium reduction during the trial could directly reflect the effect of the multiple health communication interventions. In addition, the self-monitoring of seven day salt intake as another means of intervention enabled participants to estimate their daily intake of salt, which proved useful in keeping the participants engaged in salt reduction practice and provided a useful guide to reduce salt intake to the recommended level.

### Comparison with other studies

Our results showed that the reduction in urinary sodium excretion was accompanied by a decrease in blood pressure, especially systolic blood pressure. A recent meta-analysis of randomised salt reduction trials found that every 100 mmol/day (2.3 g/day) reduction in urinary sodium excretion was associated with a 5.56 (95% confidence interval −4.52 to −6.59) mm Hg lower mean systolic blood pressure and a 2.33 (−1.66 to −3.00) mm Hg lower mean diastolic blood pressure.^25^ The decrease of 1.98 mm Hg in systolic blood pressure for a reduction in urinary sodium excretion of 336.8 mg (14.6 mmol) per 24 hours observed in our study was larger than that in the meta-analysis. One possible reason for this difference could be the duration of our trial, which spanned a year. By contrast, most of the randomised trials that were included in the meta-analysis lasted only for several weeks. Evidence shows that salt reduction does not reach its full effect on blood pressure over a few weeks.^26^

### Strengths and limitations of study

This was a large scale, community based salt reduction study targeting families in communities in eastern, central, and western regions of China. A stringent and standardised protocol was implemented at all study sites, with a high follow-up rate of 90%. An electronic data collection system was used to ensure high quality data collection. People who analysed the urine samples in the laboratory were blinded to whether the samples were from participants in the control or intervention group, which avoided potential bias in the sodium measurements.

Our study also had some limitations. Firstly, we used 24 hour urine sodium excretion to evaluate the daily salt intake, which is acknowledged to be the most accurate method. However, single 24 hour urine collection provided limited information on dietary salt intake compared with multiple 24 hour urine collections. Secondly, the study outcomes (that is, 24 hour urinary sodium and blood pressure) were assessed at the start and at the end of the trial, so the change in the intervention effect over time could not be observed. Thirdly, the effect of the intervention measures was evaluated as a whole, which means that the independent effect of each measure could not be determined.

### Conclusions and public health implications

This study showed that the comprehensive salt reduction interventions targeting home cooks and family members were effective in terms of both urinary sodium and blood pressure reduction. The reduction of 0.9 g/day in salt intake observed in our study seems modest; however, it has substantial public health significance in terms of preventing cardiovascular events and reducing medical costs.^27^ On the basis of a recent meta-analysis of blood pressure treatment trials,^28^ the reduction of 2 mm Hg in systolic blood pressure observed in our study would reduce the risk of stroke by 5.2% and ischaemic heart disease by 3.2%. This would prevent about 205 000 strokes and 112 000 ischaemic heart disease events every year in China if the programme were scaled up across the country.^29^

In 2016 the Chinese government issued its “Healthy China 2030” plan and set a goal of a 20% reduction of daily salt intake in adults by 2030. Our study was a positive action to help in achieving China’s salt reduction target, and the effect was evident during the one year trial. When implementing the interventions in the real world in the long term, strong policy support and continuing input of grassroots personnel are important guarantees for the sustainability of the community based salt reduction interventions. Village doctors and neighbourhood committees are involved in mobilising residents to participate in the activities. Grassroots professional staff are responsible for giving lectures and providing guidance on salt monitoring. Integration of the salt reduction interventions into health policy and introduction of a system of incentives and assessment for the local personnel would be useful aids to implementation. Furthermore, community based interventions should be accessible to older people, who may not be familiar with the use of a smartphone. This would make the generalisation of self-monitoring of salt intake using the WeChat app challenging.

To evaluate the long term effect of the interventions, we have been collecting data on urine sodium and blood pressure of the participants one and two years after the trial. We hope that these data will provide more evidence on the effect of the community based interventions in the future.

In conclusion, this study provides new scientific evidence on the involvement of home cooks in salt reduction interventions and has the potential to be widely applied in China and other countries where home cooking is a major source of salt intake. Feasible public health policy is needed to facilitate the sustainability of the community based salt reduction interventions.

## What is already known on this topic

Salt intake in China is more than double the recommended limit, which causes a heavy disease burden
Salt reduction is a cost effective public health policy
Most dietary salt in China is added during home cooking, but evidence from randomised controlled trials of interventions targeting home cooks has been lacking

## What this study adds

A package of community based salt reduction interventions for home cooks and family members to gain knowledge of and develop skills in salt reduction has been developed
These interventions were feasible and effective in improving salt reduction behaviours and reducing urinary sodium excretion and blood pressure
This intervention package has the potential to be scaled up to larger areas so as to help in the prevention of cardiovascular diseases

## Data Availability

Relevant anonymised individual level data will be made available one year after publication of the primary manuscript on request from the corresponding authors. Request for data sharing will be handled in line with the relevant regulations for data access and sharing in China and will need the approval of the trial steering committee and the Institutional Review Board of the Chinese Center for Disease Control and Prevention.

## Web extra

Extra material supplied by authors

Web appendix: Supplementary materials

## Notes

Contributors: PZ, XZ, JW, YL, JM, FJH, and GAM designed the study, developed the protocol, and were involved in the interpretation of the results. WY, YG, DJ, YD, FG, SY, XZ, DS, and YL were responsible for the onsite field investigation and participated in monitoring and supervision of data collection. DS, XZ, and JS were involved in the statistical analysis of the study. XZ and DS drafted the manuscript, and all authors commented on and contributed to the final version. JW and PZ received the funding and are the guarantors. PZ is the co-corresponding author and can be reached at zpuhong@georgeinstitute.org.cn. The corresponding authors attest that all listed authors meet authorship criteria and that no others meeting the criteria have been omitted.

The lead authors (the manuscript’s guarantors) affirm that the manuscript is an honest, accurate, and transparent account of the study being reported; that no important aspects of the study have been omitted; and that any discrepancies from the study as planned (and, if relevant, registered) have been explained.

Dissemination to participants and related patient and public communities: Once the study has been published, provincial Centers for Disease Control and Prevention, county level Centers for Disease Control and Prevention, primary healthcare centres, and participants will be informed of the results via newsletters, WeChat, and website updates.

Provenance and peer review: Not commissioned; externally peer reviewed.

## References

[1] Cook NR Cutler JA Obarzanek E . Long term effects of dietary sodium reduction on cardiovascular disease outcomes: observational follow-up of the trials of hypertension prevention (TOHP). BMJ 2007;334:885-8. 10.1136/bmj.39147.604896.55 17449506 PMC1857760

[2] Gardener H Rundek T Wright CB Elkind MS Sacco RL . Dietary sodium and risk of stroke in the Northern Manhattan study. Stroke 2012;43:1200-5. 10.1161/STROKEAHA.111.641043 22499576 PMC3347890

[3] He FJ MacGregor GA . Reducing population salt intake worldwide: from evidence to implementation [J]. Prog Cardiovasc Dis 2010;52:363-82. 10.1016/j.pcad.2009.12.006 20226955

[4] GBD 2019 Risk Factors Collaborators . Global burden of 87 risk factors in 204 countries and territories, 1990-2019: a systematic analysis for the Global Burden of Disease Study 2019. Lancet 2020;396:1223-49. 10.1016/S0140-6736(20)30752-2 33069327 PMC7566194

[5] Zhou M Wang H Zeng X . Mortality, morbidity, and risk factors in China and its provinces, 1990-2017: a systematic analysis for the Global Burden of Disease Study 2017. Lancet 2019;394:1145-58. 10.1016/S0140-6736(19)30427-1 31248666 PMC6891889

[6] World Health Organization. Diet, nutrition and the prevention of chronic diseases: report of a joint WHO/FAO expert consultation (WHO Technical Report Series 916). 2003. https://www.who.int/publications/i/item/924120916X. 12768890

[7] Tan M He FJ Wang C MacGregor GA . Twenty-four-hour urinary sodium and potassium excretion in China: A systematic review and meta-analysis. J Am Heart Assoc 2019;8:e012923. 10.1161/JAHA.119.012923 31295409 PMC6662145

[8] Anderson CA Appel LJ Okuda N . Dietary sources of sodium in China, Japan, the United Kingdom, and the United States, women and men aged 40 to 59 years: the INTERMAP study. J Am Diet Assoc 2010;110:736-45. 10.1016/j.jada.2010.02.007 20430135 PMC4308093

[9] Brown IJ Tzoulaki I Candeias V Elliott P . Salt intakes around the world: implications for public health. Int J Epidemiol 2009;38:791-813. 10.1093/ije/dyp139 19351697

[10] Jin A Xie W Wu Y . Effect of salt reduction interventions in lowering blood pressure in Chinese populations: a systematic review and meta-analysis of randomised controlled trials. BMJ Open 2020;10:e032941. 10.1136/bmjopen-2019-032941 32071177 PMC7044858

[11] Xu A Ma J Guo X . Association of a province-wide intervention with salt intake and hypertension in Shandong province, China, 2011-2016. JAMA Intern Med 2020;180:877-86. 10.1001/jamainternmed.2020.0904 32338717 PMC7186913

[12] Hou L Guo X Zhang J . Associations between salt-restriction spoons and long-term changes in urinary Na+/K+ ratios and blood pressure: Findings from a population-based cohort. J Am Heart Assoc 2020;9:e014897. 10.1161/JAHA.119.014897 32674645 PMC7660739

[13] Cogswell ME Loria CM Terry AL . Estimated 24-Hour Urinary Sodium and Potassium Excretion in US Adults. JAMA 2018;319:1209-20. 10.1001/jama.2018.1156 29516104 PMC5885845

[14] Jin A Xie W Wu Y . Effect of salt reduction interventions in lowering blood pressure in Chinese populations: a systematic review and meta-analysis of randomised controlled trials. BMJ Open 2020;10:e032941. 10.1136/bmjopen-2019-032941 32071177 PMC7044858

[15] Zhang X Hu X Ma J . Cluster randomised controlled trial of home cook intervention to reduce salt intake in China: a protocol study. BMJ Open 2020;10:e033842. 10.1136/bmjopen-2019-033842 32385058 PMC7228508

[16] He FJ Wu Y Feng XX . School based education programme to reduce salt intake in children and their families (School-EduSalt): cluster randomised controlled trial. BMJ 2015;350:h770. 10.1136/bmj.h770 25788018 PMC4364292

[17] Guidelines for Prevention and Treatment of Hypertension in China. (2018 Revised edition). Chinese Journal of Cardiovascular Sciences 2019;24(01):24-56.

[18] He FJ Wu Y Ma J . A school-based education programme to reduce salt intake in children and their families (School-EduSalt): protocol of a cluster randomised controlled trial. BMJ Open 2013;3:e003388. 10.1136/bmjopen-2013-003388 23864214 PMC3717470

[19] Huang L Crino M Wu JH . Mean population salt intake estimated from 24-h urine samples and spot urine samples: a systematic review and meta-analysis. Int J Epidemiol 2016;45:239-50. 10.1093/ije/dyv313 26796216

[20] Barberio AM Sumar N Trieu K . Population-level interventions in government jurisdictions for dietary sodium reduction: a Cochrane Review. Int J Epidemiol 2017;46:1551-1405. 10.1093/ije/dyw361 28204481 PMC5837542

[21] Christoforou A Trieu K Land MA Bolam B Webster J . State-level and community-level salt reduction initiatives: a systematic review of global programmes and their impact. J Epidemiol Community Health 2016;70:1140-50. 10.1136/jech-2015-206997 27222501

[22] Ma Y Feng X Ma J . Social support, social network and salt-reduction behaviours in children: a substudy of the School-EduSalt trial. BMJ Open 2019;9:e028126. 10.1136/bmjopen-2018-028126 31203245 PMC6589018

[23] Takada T Imamoto M Fukuma S . Effect of cooking classes for housewives on salt reduction in family members: a cluster randomized controlled trial. Public Health 2016;140:144-50. 10.1016/j.puhe.2016.07.005 27523782

[24] Ghimire K Adhikari TB Rijal A Kallestrup P Henry ME Neupane D . Knowledge, attitudes, and practices related to salt consumption in Nepal: Findings from the community-based management of non-communicable diseases project in Nepal (COBIN). J Clin Hypertens (Greenwich) 2019;21:739-48. 10.1111/jch.13544 31026125 PMC8030483

[25] Filippini T Malavolti M Whelton PK Naska A Orsini N Vinceti M . Blood pressure effects of sodium reduction: Dose-response meta-analysis of experimental studies. Circulation 2021;143:1542-67. 10.1161/CIRCULATIONAHA.120.050371 33586450 PMC8055199

[26] He FJ MacGregor GA . Role of salt intake in prevention of cardiovascular disease: controversies and challenges. Nat Rev Cardiol 2018;15:371-7. 10.1038/s41569-018-0004-1 29713009

[27] Bibbins-Domingo K Chertow GM Coxson PG . Projected effect of dietary salt reductions on future cardiovascular disease. N Engl J Med 2010;362:590-9. 10.1056/NEJMoa0907355 20089957 PMC3066566

[28] Blood Pressure Lowering Treatment Trialists’ Collaboration . Pharmacological blood pressure lowering for primary and secondary prevention of cardiovascular disease across different levels of blood pressure: an individual participant-level data meta-analysis. Lancet 2021;397:1625-36. 10.1016/S0140-6736(21)00590-0 33933205 PMC8102467

[29] GBD 2019 Diseases and Injuries Collaborators . Global burden of 369 diseases and injuries in 204 countries and territories, 1990-2019: a systematic analysis for the Global Burden of Disease Study 2019. Lancet 2020;396:1204-22. 10.1016/S0140-6736(20)30925-9 33069326 PMC7567026

