# Supplementary material for: Effect of home cook interventions for salt reduction in China: cluster randomised controlled trial
Source: BMJ. 2023 Aug 24;382:e074258. doi: 10.1136/bmj-2022-074258 (PMC10448250; doi:10.1136/bmj-2022-074258)
Supplement: Supplementary file 1 — Web appendix: Supplementary materials [file zhax074258.ww1.pdf]

Supplementary Material

Supplementary Table A. Composition of the intervention package and implementation plan

Intervention components	Description and implementation
**Environment building**	
Posters	Five different kinds of posters were provided for each community
Short videos	Three short videos were provided and were asked to play for 60 min per day at each primary health institute which the intervention community is under jurisdiction
Loudspeaker broadcast	Eight loudspeaker broadcasts were provided for each community and were asked to play 30 min per day at each primary health institute which the intervention community is under jurisdiction
Leaflets	Three different kinds of leaflets were provided for each community
Manual	One manual was distributed for each community
**Lectures**	Once two months, 40 min each time, given by CDC at county level
**Family salt intake monitoring**	Once two months, instructed by CDC at the county level

Supplementary Table B. Characteristics of participants followed and lost to follow-up at the end of one-year intervention.

Characteristics	Control group				Intervention group			
	Followed (n, %)	Lost (n, %)	χ^2	P value	Followed (n, %)	Lost (n, %)	χ^2	P value
**Total**	713(100.00)	77(100.00)			706(100.00)	80(100.00)		
**Gender**			1.997	0.158			0.474	0.49
Male	347(48.67)	44(57.14)			342(48.44)	42(52.50)		
Female	366(51.33)	33(42.86)			364(51.56)	38(47.50)		
**Age (year)**			11.34	0.001			5.146	0.02
18-59	435(61.01)	62(80.52)			401(56.80)	56(70.00)		
60-75	278(38.99)	15(19.48)			305(43.20)	24(30.00)		
**Education level**			10.854	0.013			1.842	0.61
Never Enrolled in Education	170(23.84)	7(9.09)			156(22.10)	13(16.25)		
Primary school	209(29.31)	23(29.87)			217(30.74)	29(36.25)		
Junior high school	230(32.26)	29(37.66)			210(29.75)	24(30.00)		
High school and above	104(14.59)	18(23.38)			123(17.42)	14(17.50)		
**Total annual household income (Yuan)** [a]			10.863	0.001			1.098	0.29
≤25000	341(54.21)	23(33.33)			343(54.36)	37(48.05)		
＞25000	288(45.79)	46(66.67)			288(45.64)	40(51.95)		
**Drinker**			4.288	0.038			5.778	0.02
No	456(63.96)	40(51.95)			450(63.74)	40(50.00)		
Yes	257(36.04)	37(48.05)			256(36.26)	40(50.00)		
**Achieve moderate and above intensity exercise**			2.882	0.090			4.159	0.04
Yes	544(76.30)	52(67.53)			533(75.50)	52(65.00)		
No	169(23.70)	25(32.47)			152(21.53)	28(35.00)		
**BMI** (kg/m^2), Mean (SD)	24.77(3.56)	28.30(21.60)	2.811[a]	0.005	25.16(3.73)	24.91(4.01)	-0.981[b]	0.33
**Outdoor temperature (℃)**, Mean (SD)	7.81(13.57)	8.89(13.23)	-0.215[a]	0.830	12.10(6.88)	12.31(6.93)	0.374[b]	0.71

[a] There were missing data. [b] rank sum test was used.

Supplementary Table C. Impact of intervention on systolic blood pressure (mmHg) by subgroup

Characteristics	Control					Intervention					Difference of changes between groups (95% CI) [b,c]	P value	P for interaction
	Baseline		12-month		Change (95% CI) [a,c]	Baseline		12-month		Change (95% CI) [a,c]			
	N	Mean (SD)	N	Mean (SD)		N	Mean (SD)	N	Mean (SD)				
**Gender**													0.49
Male	391	131.1(17.8)	347	133.4(19.0)	0.5(-1.1 to 2.1)	384	132.1(17.7)	342	132.5(17.2)	-2.0(-3.8 to -0.2)	-2.5(-4.7 to -0.3)	0.02	
Female	399	126.8(20.0)	366	127.7(18.9)	-0.7(-2.3 to 0.8)	401	132.1(17.7)	364	132.5(17.2)	-2.2(-4.0 to -0.5)	-1.5(-3.6 to 0.7)	0.18	
**Age (year)** [d]													0.84
18-49.1	233	121(1.4)	165	122(1.6)	0.92(-1.6 to 3.4)	191	122(1.6)	152	121(1.7)	-0.72(-3.5 to 2.1)	-0.27(-4.2 to 3.7)	0.89	
49.2-56.4	208	128(1.2)	184	130(1.2)	1.58(-0.3 to 3.5)	200	127(1.3)	158	127(1.3)	-0.47(-2.8 to1.9)	-2.14(-5.2 to 0.9)	0.16	
56.5-65.3	180	134(1.2)	178	135(1.2)	0.08(-1.9 to 2.0)	206	134(1.2)	191	133(1.2)	-1.47(-3.6 to 0.7)	-0.76(-3.7 to 2.2)	0.61	
65.4-75	169	136(1.4)	186	136(1.4)	0.53(-1.8 to 2.8)	189	136(1.4)	205	137(1.3)	0.68(-1.6 to 3.0)	0.62(-2.8 to 4.1)	0.72	
**Education level**													0.88
Primary and below	409	131.6(19.5)	385	132.5(19.0)	-0.3(-1.9 to 1.3)	414	132.5(19.3)	374	132.6(17.9)	-2.2(-3.9 to -0.4)	-1.9(-4.0 to 0.2)	0.08	
Junior high and above	381	126.0(18.2)	328	128.1(19.0)	0.03(-1.6 to 1.7)	371	127.0(18.6)	332	127.1(18.0)	-2.1(-3.9 to -0.3)	-2.1 (-4.4 to 0.2)	0.07	
**Family income (Yuan)**													0.63
≤25000	364	131.9(19.9)	293	134.4(19.5)	-0.2(-2.0 to 1.6)	379	132.4(19.7)	315	132.2(18.0)	-2.3(-4.4 to -0.3)	-2.1(-4.7 to 0.4)	0.10	
>25000	334	126.5(18.0)	401	127.4(18.4)	-1.5(-1.9 to 1.6)	328	126.6(18.2)	368	128.3(18.2)	-1.4(-3.3 to 0.5)	-1.3(-3.7 to 1.2)	0.31	
**Hypertension**													0.40
Yes	236	145.2(17.1)	241	145.3(17.2)	-0.8(-2.4 to 0.9)	259	144.1(16.6)	238	143.0(15.6)	-2.0(-3.7 to -0.3)	-1.3(-3.5 to 1.0)	0.27	
No	554	118.4(11.4)	472	119.2(11.6)	0.2(-1.2 to 1.6)	526	117.6(11.1)	468	119.1(12.0)	0.2(-1.4 to 1.8)	0.04(-1.9 to 2.0)	0.97	
**Home cook**													0.83
Yes	395	127.2(19.5)	363	128.7(19.1)	-0.2(-1.7 to 1.4)	392	129.0(19.5)	356	129.0(18.6)	-2.3(-4.1 to -0.6)	-2.2(-4.3 to 0.0)	0.05	
No	395	130.7(18.5)	350	132.3(19.0)	-0.1(-1.7 to 1.5)	393	130.7(18.8)	350	131.1(17.7)	-1.9(-3.7 to -0.2)	-1.8(-4.0 to 0.4)	0.10	

[a] Comparison of the means between baseline and 12-month follow-up. Positive values indicate increases from baseline and negative values indicate decreases.

[b] Comparison of changes from baseline to 12-month between groups. Negative values support the effect of intervention on lowering systolic blood pressure, while positive values oppose it.

[c] Adjusted for age, sex, BMI, city and education level.

[d] Age was defined as natural cubic spline with internal knots at 25th, 50th and 75th percentiles.

Supplementary Table D. Impact of intervention on diastolic blood pressure (mmHg) by subgroup

Characteristics	Control					Intervention					Difference of changes between groups (95% CI) [b,c]	P value	P for interaction
	Baseline		12-month		Change (95% CI) [a,c]	Baseline		12-month		Change (95% CI) [a,c]			
	N	Mean (SD)	N	Mean (SD)		N	Mean (SD)	N	Mean (SD)				
**Gender**													0.74
Male	391	81.7(10.8)	347	82.0(11.5)	-0.2(-1.2 to 0.8)	384	81.7(11.1)	342	81.3(10.9)	-1.1(-2.2 to -1.8)	-0.9(-2.2 to 0.4)	0.19	
Female	399	78.4(10.7)	366	78.2(10.3)	-0.6(-1.5 to 0.4)	401	78.6(11.3)	364	77.4(11.3)	-1.8(-2.9 to -0.7)	-1.2(-2.5 to 0.1)	0.07	
**Age (year)** [d]													0.80
18-49.1	233	78.1(0.9)	165	78.9(1.0)	0.76(-0.8 to 2.3)	191	78.8(1.0)	152	79.5(1.1)	0.69(-1.0 to 2.4)	0.68(-1.8 to 3.1)	0.58	
49.2-56.4	208	81.7(0.7)	184	82.4(0.8)	0.72(-0.5 to 1. 9)	200	80.6(0.8)	158	80.9(0.8)	0.29(-1.2 to 1.7)	-1.29(-3.2 to 0.6)	0.18	
56.5-65.3	180	81.6(0.7)	178	81.7(0.8)	0.16(-1.0 to 1.4)	206	81.4(0.7)	191	80.7(0.7)	-0.74(-2.1 to 0.6)	-0.591(-2.4 to 1.3)	0.53	
65.4-75	169	79.0(0.9)	186	78.5(0.9)	-0.51(-1.9 to 0.9)	189	78.5(0.9)	205	78.3(0.8)	-0.25(-1.7 to 1.2)	-0.32(-2.5 to 1.8)	0.76	
**Education level**													0.06
Primary and below	409	80.1(10.8)	385	79.8(10.9)	-0.3(-1.2 to 0.7)	414	79.9(11.1)	374	78.3(11.0)	-2.2(-3.2 to -1.1)	-1.9(-3.2 to -0.6)	0.004	
Junior high and above	381	80.0(10.8)	328	80.4(11.3)	-0.5(-1.5 to 0.5)	371	80.3(11.5)	332	80.4(11.5)	-0.5(-1.7 to 0.6)	-0.1(-1.5 to 1.3)	0.92	
**Family income (Yuan)**													0.13
≤25000	364	80.1(10. 8)	293	80.2(11.4)	-0.5(-1.6 to 0.6)	379	80.3(11.2)	315	78.6(11.3)	-2.4(-3.7 to -1.2)	-1.9(-3.5 to -0.4)	0.02	
>25000	334	80.3(10.6)	401	79.8(10.8)	-0.2(-1.3 to 0.8)	328	80.0(11.7)	368	80.0(11.3)	-0.5(-1.6 to 0.7)	-0.2(-1.7 to 1.3)	0.78	
**Hypertension**													0.44
Yes	236	87.6(10.9)	241	86.6(11.1)	-1.0(-2.0 to 0.1)	259	86.8(10.7)	238	85.5(11.4)	-1.6(-2.7 to -0.6)	-0.7(-2.1 to 0.7)	0.35	
No	554	75.2(7.6)	472	75.1(8.0)	0.0(-0.8 to 0.9)	526	74.1(7.9)	468	74.2(8.2)	0.1(-0.9 to 1.1)	-0.1(-1.2 to 1.3)	0.91	
**Home cook**													0.59
Yes	395	78.8(10.4)	363	78.7(10.6)	-0.4(-1.4 to 0.6)	392	79.3(11.2)	356	78.2(11.7)	-1.7(-2.8 to -0.6)	-1.3(-2.6 to 0.0)	0.05	
No	395	81.3(11.1)	350	81.4(11.3)	-0.4(-1.3 to 0.6)	393	80.9(11.3)	350	80.4(10.7)	-1.2(-2.2 to -0.1)	-0.8(-2.1 to 0.5)	0.24	

[a] Comparison of the means between baseline and 12-month follow-up. Positive values indicate increases from baseline and negative values indicate decreases.

[b] Comparison of changes from baseline to 12-month between groups. Negative values support the effect of intervention on lowering diastolic blood pressure, while positive values oppose it.

[c] Adjusted for age, sex, BMI, city and education level.

[d] Age was defined as natural cubic spline with internal knots at 25th, 50th and 75th percentiles.

Supplementary Table E. Sensitive analysis without adjusting confounders.

Outcomes	Control					Intervention					Difference of changes between groups (95% CI) [b,c]	P value
	Baseline		12-month		Change (95% CI) [a,c]	Baseline		12-month		Change from baseline (95% CI) [a,c]		
	N	Mean (SD)	N	Mean (SD)		N	Mean (SD)	N	Mean (SD)			
**Urinary sodium(mg/24h)**	769	4418.7 (1973.7)	678	4330.9 (1859.8)	-90.4 (-237.4 to 56.7)	761	4368.7 (1880.3)	667	3977.0 (1688.8)	-408.8 (-556.8 to-260.7)	-318.4 (-527.0 to -109.7)	0.003
**Urinary potassium(mg/24h)**	769	1578.5 (617.0)	678	1513.0 (598.6)	-73.2 (-124.6 to -21.7)	761	1593.8 (616.3)	667	1542.4 (599.0)	-56.5 (-108.3 to -4.7)	16.7 (-56.3 to 89.7)	0.65
**Sodium-to-potassium Ratio(mmol/mmol)**	769	5.1(2.2)	678	5.3(2.3)	0.3(0.1 to 0.4)	761	5.0(2.2)	667	4.7(2.1)	-0.2(-0.4 to 0)	-0.5(-0.7 to -0.2)	<0.001
**SBP (mmHg)**	790	128.9(19.1)	713	130.5(19.2)	1.3(0.3 to 2.4)	785	130.0(19.1)	706	130.1(18.2)	-0.01(-1.1 to 1.1)	-1.3(-2.8 to 0.2)	0.09
**DBP (mmHg)**	790	80.1(10.8)	713	80.1(11.0)	-0.1(-0.7 to 0.6)	785	80.1(11.3)	706	79.3(11.3)	-0.8(-1.5 to -0.1)	-0.7(-1.7 to 0.2)	0.12

[a] Comparison of the means between baseline and 12-month follow-up. Positive values indicate increases from baseline and negative values indicate decreases.

[b] Comparison between the intervention group and the control group in the changes from baseline to 12-month follow-up. Positive values indicate that the intervention group has a greater increase or less decrease from baseline to 12-month follow-up compared with the control group, and negative values indicate the intervention group has a greater decrease or smaller increase from baseline to 12-month follow-up compared with control group.

[c] Results were obtained from a mixed linear model with random intercept of participants nested within family and random intercept of family nested within communities.

Supplementary Table F. Sensitivity analysis with possibly incomplete 24-hour urine samples included and confounders adjusted.

Outcomes	Control					Intervention					Difference of changes between groups (95% CI) [b,c]	P value
	Baseline		12-month		Change (95% CI) [a,c]	Baseline		12-month		Change from baseline (95% CI) [a,c]		
	N	Mean (SD)	N	Mean (SD)		N	Mean (SD)	N	Mean (SD)			
**Urinary sodium(mg/24h)**	790	4362.2 (1992.0)	699	4271.4 (1872.7)	-56.8 (-202.8, 89.3)	782	4307.1 (1906.9)	705	3885.6 (1799.2)	-404.5 (-550.1 to -258.8)	-347.7 (-553.1 to -142.3)	0.001
**Urinary potassium(mg/24h)**	790	1561.3 (627.7)	699	1490.7 (605.0)	-80.6 (-131.5, -29.8)	782	1568.8 (628.9)	705	1499.6 (614.3)	-73.6 (-124.3 to -22.8)	7.1 (-64.5 to 78.6)	0.85

[a] Comparison of the means within each group between baseline and 12-month follow-up. Positive values indicate increases from baseline to 12-month follow-up and negative values indicate decreases from baseline to 12-month follow-up.

[b] Comparison between the intervention group and the control group in the changes from baseline to 12-month follow-up. Positive values indicate that the intervention group has a greater increase or less decrease from baseline to 12-month follow-up compared with the control group, and negative values indicate the intervention group has a greater decrease or smaller increase from baseline to 12-month follow-up compared with control group.

[c] Results were obtained from a mixed linear model with random intercept of participants nested within family and random intercept of family nested within communities, adjusted for age, sex, BMI, city and education level.
